# Blood-based kinase activity profiling: a potential predictor of response to immune checkpoint inhibition in metastatic cancer

**DOI:** 10.1136/jitc-2020-001607

**Published:** 2020-12-22

**Authors:** Daan P Hurkmans, Els M E Verdegaal, Sabrina A Hogan, Rik de Wijn, Lies Hovestad, Dianne M A van den Heuvel, Rob Ruijtenbeek, Marij J P Welters, Mandy van Brakel, Edwin A Basak, Herbert M Pinedo, Cor H J Lamers, Harmen J G van de Werken, John P Groten, Reno Debets, Mitchell P Levesque, Reinhard Dummer, Ellen Kapiteijn, Ron H J Mathijssen, Joachim G J V Aerts, Sjoerd H van der Burg

**Affiliations:** 1 Department of Pulmonology, Erasmus University Medical Center, Rotterdam, The Netherlands; 2 Department of Medical Oncology, Erasmus MC Cancer Institute, Erasmus University Medical Center, Rotterdam, The Netherlands; 3 Department of Medical Oncology, Oncode Institute, Leiden University Medical Center, Leiden, The Netherlands; 4 Department of Dermatology, University Hospital Zurich, Zurich, Switzerland; 5 PamGene International B.V, HH 's-Hertogenbosch, The Netherlands; 6 Department of Urology, Erasmus MC Cancer Institute, Erasmus University Medical Center, Rotterdam, The Netherlands; 7 Cancer Computational Biology Center, Erasmus MC Cancer Institute, Erasmus University Medical Center, Rotterdam, The Netherlands; 8 Department of Medical Oncology, Leiden University Medical Center, Leiden, The Netherlands

**Keywords:** immunotherapy, melanoma, lung neoplasms, immunity, cellular

## Abstract

**Background:**

Many cancer patients do not obtain clinical benefit from immune checkpoint inhibition. Checkpoint blockade targets T cells, suggesting that tyrosine kinase activity profiling of baseline peripheral blood mononuclear cells may predict clinical outcome.

**Methods:**

Here a total of 160 patients with advanced melanoma or non-small-cell lung cancer (NSCLC), treated with anti-cytotoxic T-lymphocyte-associated protein 4 (anti-CTLA-4) or anti-programmed cell death 1 (anti-PD-1), were divided into five discovery and cross-validation cohorts. The kinase activity profile was generated by analyzing phosphorylation of peripheral blood mononuclear cell lysates in a microarray comprising of 144 peptides derived from sites that are substrates for protein tyrosine kinases. Binary grouping into patients with or without clinical benefit was based on Response Evaluation Criteria in Solid Tumors V.1.1. Predictive models were trained using partial least square discriminant analysis (PLS-DA), performance of the models was evaluated by estimating the correct classification rate (CCR) using cross-validation.

**Results:**

The kinase phosphorylation signatures segregated responders from non-responders by differences in canonical pathways governing T-cell migration, infiltration and co-stimulation. PLS-DA resulted in a CCR of 100% and 93% in the anti-CTLA-4 and anti-PD1 melanoma discovery cohorts, respectively. Cross-validation cohorts to estimate the accuracy of the predictive models showed CCRs of 83% for anti-CTLA-4 and 78% or 68% for anti-PD-1 in melanoma or NSCLC, respectively.

**Conclusion:**

Blood-based kinase activity profiling for response prediction to immune checkpoint inhibitors in melanoma and NSCLC revealed increased kinase activity in pathways associated with T-cell function and led to a classification model with a highly accurate classification rate in cross-validation groups. The predictive value of kinase activity profiling is prospectively verified in an ongoing trial.

## Background

Tumors can evade T-cell-mediated destruction via the expression of immune checkpoints, including the programmed cell death ligand-1 (PD-L1) and CD80 or CD86, that inhibit T cells that express programmed cell death 1 (PD-1) or cytotoxic T lymphocyte antigen 4 (CTLA-4), respectively. Immune checkpoint inhibitors (ICIs) against these receptors have been approved for a variety of malignancies and revolutionized their clinical management, in particular that of melanoma and non-small cell lung cancer (NSCLC).^1–4^ However, durable responses are only obtained in a minority of patients, whereas ICIs are associated with considerable side effects and costs. Therefore, robust and reliable predictive biomarkers to predict treatment response are urgently needed.

The cognate interaction between T cells and antigen presenting cells (APCs) via interaction of the T-cell receptor (TCR) with antigen presented in the context of HLA, results in activation of T cells after which T cells quickly upregulate CTLA-4 and/or PD-1 as part of a negative feedback loop. As a result, T cells may display a reduced capacity to become activated, proliferate and exert specific effector functions. Currently, CTLA-4 is thought to play a major role during priming of a T-cell response in the lymph node where it directly prevents co-stimulation of T cells via interaction of CD28 with its ligand CD80/86 on APCs. Moreover, CTLA-4 is constitutively expressed on regulatory T cells (Tregs), and Tregs can reduce expression of CD80/86 by trans-endocytosis thereby preventing activation of effector T cells.^5^ PD-1 inhibits activation of pre-existing tumor-specific T cells during the effector phase and is thought to dampen an immune response after antigen eradication in order to prevent immune pathology. Eventually, tumor eradication by T cells relies on TCR-mediated activation and downstream co-stimulatory signaling, which is tightly regulated by different tyrosine kinase mediated signaling pathways. For instance, activation of PI3K and deactivation of PTEN results in recruitment and activation of downstream signaling molecules like AKT, enhancing T-cell survival, proliferation and effector functions.^6^ Under normal conditions, ligation of the inhibitory receptors CTLA-4 and PD-1 results in recruitment of SHP2 phosphatases that dampens TCR signaling as well as CD28 signaling.^7–9^ As such, kinase activity may reflect anti-tumor T-cell activity and consequently could act as a predictor for clinical outcome after ICI therapy. In fact, peptide microarray technology to evaluate global kinase activities in tumor or blood has recently been applied as a biomarker strategy for response prediction to chemotherapy or targeted therapy in several cancer types.^6 10–13^

Previous efforts to predict the clinical response to ICI therapy yielded various biomarkers, including tumor mutational burden (TMB) and PD-L1 expression in tumor tissue for NSCLC.^14 15^ The predictive performance of these biomarkers may be sufficient in some studies,^16 17^ yet are complicated by both the availability of tissue and intertumoral and intratumoral heterogeneity. Interestingly, a number of blood parameters have been associated with response to anti-CTLA-4 and anti-PD-1/PD-L1. For example, the total number and composition of circulating leukocytes were associated with clinical outcome, among which high lymphocyte and eosinophil counts, low monocyte count and low neutrophil to lymphocyte ratio (NLR). In addition, the absence of myeloid-derived suppressor cells and presence of classical monocytes or previously activated T cells was associated with better response rates or survival after ICI.^18–28^ Collectively, these studies indicate that the activation status and number of several immune cells in blood may provide minimally invasive predictive biomarkers that are suitable for routine clinical use.

Whereas commonly used methods, including transcriptomics and high-dimensional flow cytometry may reveal the outcome of certain incoming signals, they do not reveal the whole network of signal transduction pathways activated in cells, nor show at which point they are deregulated in certain patients. Protein kinases are a large family of highly influential proteins which modify the activity, affinity and location of many cellular proteins in order to regulate cellular processes, in particular signal transduction. Here, we argued that if some of the complex biological tumor-immune cell interactions determining the response to ICIs are also reflected in the plethora of immune cells in the blood, then kinase activity profiling of peripheral blood mononuclear cells (PBMCs) may be able to capture this. Therefore, we have explored the predictive performance of kinase activity profiling in PBMCs from advanced melanoma and NSCLC patients treated with anti-PD-1 or anti-CTLA-4 monotherapy.

## Methods

### Patient population and study workflow

Patients with irresectable stage III/IV advanced melanoma or stage IV NSCLC were included if they had received intravenous monotherapy with either ipilimumab (anti-CTLA-4; 3 mg/kg every 3 weeks for four courses), nivolumab (anti-PD-1; 3 mg/kg every 2 weeks until a maximum of 2 years) or pembrolizumab (anti-PD-1; 2 mg/kg every 3 weeks until a maximum of 2 years) as standard of care at the Erasmus University Medical Center (Rotterdam, The Netherlands), Leiden University Medical Center (Leiden, The Netherlands) or University Hospital Zürich (Zürich, Switzerland), all being referral hospitals. Patients who received ICI combination therapy or who were treated with a prior line of any form of immunotherapy were excluded, pretreatment with corticosteroids was not considered. Following written informed consent of the patients, blood samples were collected before the first administration of ICIs and after the last administration of any previous treatment if applicable.

### Data collection

Binary grouping was performed according to patients with (responders) or without (non-responders) clinical benefit based on Response Evaluation Criteria in Solid Tumors V.1.1. For determination of best overall response (BOR), confirmation of a complete or partial response (CR/PR) was not required, but a minimum duration of 90 days was required for stable disease (SD). Patients with a CR, PR or SD as BOR were considered to have obtained clinical benefit after ICIs and were defined as responders. Patients with progressive disease (PD) were defined as non-responders. Additionally, binary grouping was performed according to progression-free survival, measured from start of treatment to death or the first evaluation time point that PD is detected; the two groups included late (>140 days) or no progression (responders) versus patients with early progression within 140 days (non-responders). Clinical parameters and chemistry or blood parameters were evaluated at baseline and included age, gender, WHO performance score, pathological tumor type, presence of brain metastases and serum lactate dehydrogenase (LDH) levels.

### Preparation of PBMC lysate

Venous blood of patients was collected at baseline using either sodium-heparin or EDTA as anticoagulant, and isolation of PBMCs was done within 4 hours or within 24 hours depending on the local study protocol (online supplemental table 1). PBMC were isolated by density gradient centrifugation and cryopreserved until further use. Erythrocyte lysis was only performed in the *Mel-CTLA4-B* cohort if PBMC still contained considerable erythrocyte contamination after isolation. Importantly, kinase activity was affected by erythrocyte lysis during PBMC isolation (online supplemental figure 1a). Moreover, overall reduced kinase activity was observed in PBMCs that were isolated after 24 hours from blood collected in EDTA anticoagulated tubes (online supplemental figure 1b).

10.1136/jitc-2020-001607.supp1 Supplementary data

10.1136/jitc-2020-001607.supp2 Supplementary data

Cryopreserved cells were thawed, washed with phosphate-buffered saline and lysed using ice-cold M-PER lysis buffer (Mammalian Protein Extraction Reagent, Thermo Fisher Scientific, Massachusetts, USA) containing 1:100 Protease Inhibitor Cocktail (Thermo Fisher Scientific). Cell lysate was obtained by centrifugation at 14.000 ×*g* for 10 min at 4°C. After centrifugation, the supernatant was snap-frozen in aliquots and stored at −80°C. The protein concentration was determined using the Bradford assay (Thermo Fisher Scientific) with bovine serum albumin (BSA) as the standard.

### Kinase activity profiling

PBMC kinomic activity was measured using protein tyrosine kinase (PTK) PamChip-96 microarrays (catalog # 86311, PamGene International BV, ’s-Hertogenbosch, The Netherlands) using standard manufacturer protocol. In short, the microarrays were blocked with 2% BSA (Calbiochem # 126609) to prevent non-specific binding. After blocking, the arrays were washed three times with 1×PK buffer (50 mM Tris-HCl pH 7.5, 10 mM MgCl_2_, 0.01% Brij35, 2 mM DTT). For kinomic profiling 2 µg PBMC lysate protein was used in 40 µL PTK assay buffer (1×PK buffer, 10mM DTT, 400 µM ATP, 1×PTK additive (PamGene International BV), 1:400 Halt Phosphatase Inhibitors (Thermo Fisher Scientific), 0.01% BSA (Calbiochem), and fluorescein isothiocyanate-labeled antiphosphotyrosine antibody (PamGene). During incubation, the reaction mixture was pumped up and down through the porous membrane for 60 cycles at 2 cycles/min. Incubation and read-out of the microarrays was performed with 96 arrays in parallel on a PamStation-96. Typically, three to four technical replicates of each sample were measured in the same run. Time courses of peptide phosphorylation were followed by recording fluorescent images which were quantified by automated image analysis in Bionavigator 6.3.67 (PamGene). Analysis of signals was performed after local background subtraction in Bionavigator 6.3.67 interfaced to the open source statistical program R 3.3.1 (R-project, www.rproject.org).

Analysis was specifically performed per cohort and comprised a quality check, removal of outlier replicates, data transformation and averaging of the kinase signal replicates. To check data quality, the first step was exclusion of a low portion of arrays that showed clear visual defects (eg, broken membrane and large stains) or technical replicates clearly deviating from the other replicates of the same samples. In the second step, data was log-transformed (CTLA-4 cohorts: *Mel-CTLA4-A*, *Mel-CTLA4-B*) or normalized using the variance-stabilizing normalization (VSN) method (PD-1 cohorts: *Mel-PD1-A*, *Mel-PD1-B*, *NSCLC-PD1*). Prior to log-transformation, a small fraction of negative values in the (background corrected) signals was handled by setting all signals<1 or equal to 1. Signal-positive spots were required to show a positive trend in the recorded phosphorylation time course. Peptides for which such a trend could not be detected in >75% of the samples were excluded from further analysis. Effectively, 88 to 113 peptides were included for further analysis. Technical replicates of each patient sample were averaged resulting in a single kinase activity profile per included patient for subsequent analysis.

Principal component analysis (PCA) was performed on the transformed and filtered data and PCA scores were used to identify systematic variation, for example, as a result of different sub-cohorts and/or the use of different PamStation-96 microarray plates. Systematic variation was handled by applying ComBat^29^ batch correction, and outlier patient samples were removed. For the small *Mel-CTLA4-A* cohort, no such correction was necessary. For the *Mel-CTLA4-B* cohort, a batch effect was observed and corrected for the batches in which samples were lysed together. A small number (ranging from one to eight) of outlier samples according to PCA scores or heat map visualizations of the transformed profiles were removed. In most cases, these were samples showing low overall kinase activity. Other reasons for removing measured patient samples from the analysis was unavailability of clinical data or if, after re-evaluation, patients were observed not to fulfill the selection criteria.

### Statistical analysis and bioinformatics

Kinase activity profiles were correlated to the clinical response to ICIs. Univariate (per peptide) analysis was performed using a two-sided two sample t-test with binary grouping as a covariate (responders vs non-responders). Peptides with p<0.05 were regarded as significant; in addition, the proportion of false discoveries in a set of significant peptides was estimated using the false discovery rate (FDR) method of Benjamini and Hochberg. Classification analysis was performed using partial least square discriminant analysis (PLS-DA) with patients divided in binary groups as previously described.^13^ In short, a classification model is trained using all tested peptides, that is, without prior peptide selection based on responder and non-responder differences. The resulting model is a set of coefficients (one for each peptide+an offset) that can be applied to new observations to obtain a score that predicts classification in either groups. For each cohort, the correct classification rate (CCR) was estimated using cross validation. Approximately 90% binomial CIs (CI_90_) for the CCR estimates were obtained using the exact method (note that a CI_90_ implies with 95% confidence that the CCR is higher than the lower limit). Prediction of kinases responsible for the changes in peptide phosphorylation in the kinomic profiles were obtained using the Upstream Kinase Analysis tool in Bionavigator 6.3.67 (PamGene). Results were visualized by annotation to a kinase phylogenetic tree using the web-based Coral tool (http://phanstiel-lab.med.unc.edu).^30^ Additionally, Ingenuity Pathway Analysis (Qiagen, Hilden, Germany) was used to perform gene set enrichment analyzes using the delta and unadjusted p value of each peptide (two-sided two sample t-tests). Pathway activation or inhibition were predicted by the z-score statistic, and further explored by the MAP tool (Qiagen, Hilden, Germany). Full access of the data sets generated during the current study is provided in the supplementary materials (online supplemental tables 2-6).

10.1136/jitc-2020-001607.supp5 Supplementary data

## Results

**Figure 1:**
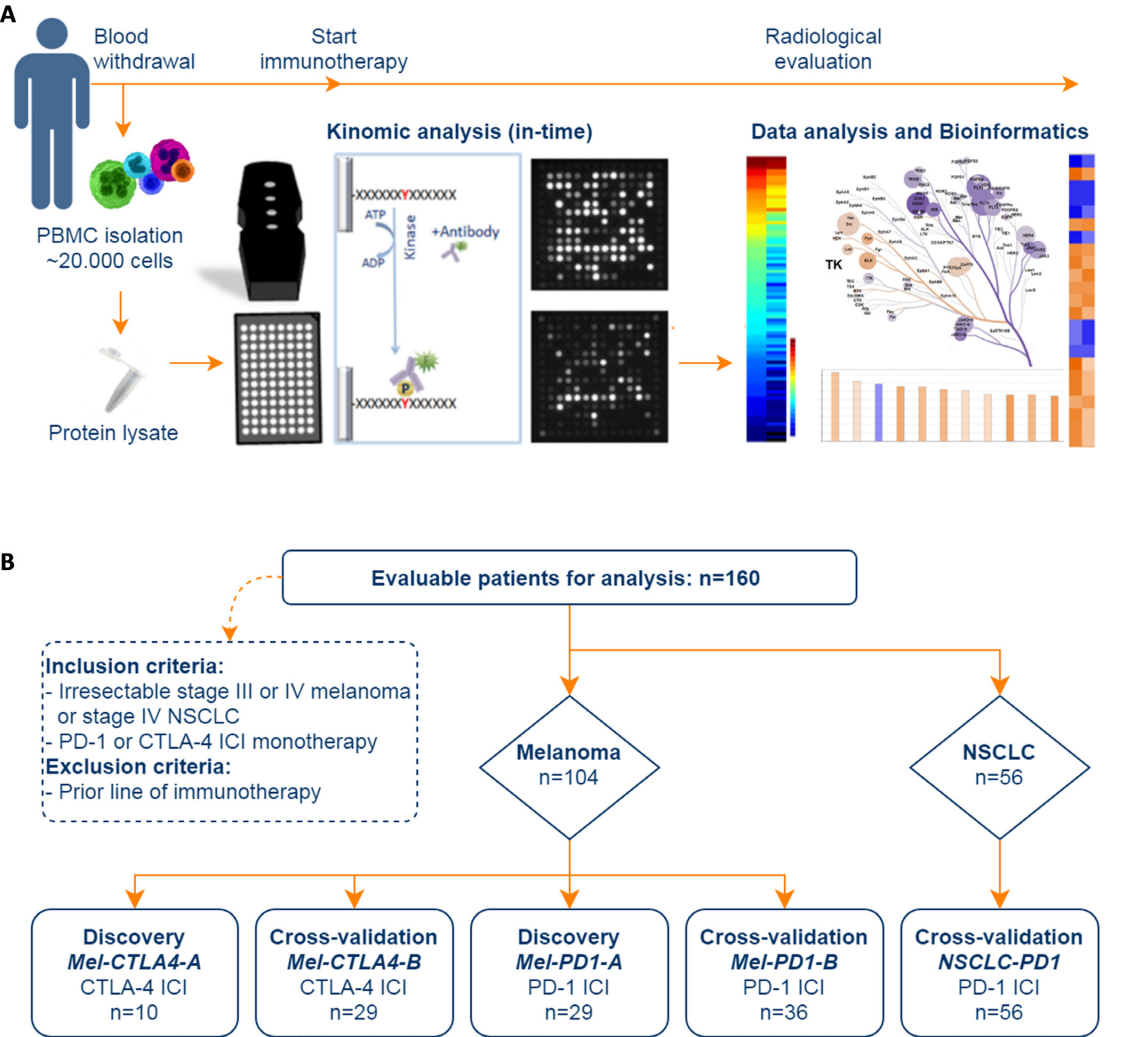
Schematic overview of the study. (A) Work flow showing that kinase activity was measured in baseline PBMC samples using a peptide microarray system consisting of identical arrays, each containing 144 unique protein tyrosine kinase phosphorylation sites. PBMC samples were isolated from blood collected before onset of ICI therapy. The kinase activity profile was analyzed using BioNavigator (PamGene), Coral tool (courtesy Cell Signaling Technologies) to annotate kinases onto a phylogenetic tree and ingenuity pathway analysis software (Qiagen) to perform gene set enrichment analysis. (B) Flow chart of the patient selection process. ICI, immune checkpoint inhibitor; NSCLC, non-small cell lung cancer; PBMC, peripheral blood mononuclear cells; PD-1, programmed cell death 1

A total of 174 advanced cancer patients were enrolled and 160 patients were evaluable for analysis (figure 1) because in 14 cases (8%), the patient samples had to be removed as they did not comply with the quality check (online supplemental figure 2). The study protocols between the centers were different with regard to the PBMC isolation protocol and the anti-coagulant used for blood collection. As a consequence, the different patient cohorts were not pooled for analysis, resulting in five distinct cohorts (figure 1), and protocol differences could be assessed for their effect on kinase activities. The baseline patient characteristics are provided in table 1. Patient cohort *Mel-CTLA4-A* was used as a discovery cohort for anti-CTLA-4 and consists of 10 melanoma patients selected based on an equal distribution of responders and non-responders. All responders in this cohort had SD >90 days, thus precluding selection bias based on exceptionally good and worse responders, only. The patients had a baseline blood LDH level that was not above 2× the upper limit of normal (ULN, ie, <250 U/mL) to avoid bias towards a known independent prognostic factor. The 29 patients in cohort *Mel-PD1-A* functioned as discovery cohort for anti-PD-1 and had an almost equal distribution of responders (n=14) and non-responders (n=15). The mean baseline blood LDH level was not elevated above 2× ULN in the *Mel-PD1-A* discovery cohort, although some individual patients did have an elevated LDH above this threshold. The patients in the three other cohorts were used for cross-validation to assess the performance of response prediction by kinase activity profiling. Because of the lower response rate, patients in the *MEL-CTLA4-B* cohort were selected on equal distribution of responsers and non-responders, but again not based on patients with an exceptionally good or bad response. Patients in the other cohorts were not selected, apart from the exclusion criteria that they should not have received prior immunotherapy or combination checkpoint blockade. The mean baseline blood LDH level was higher and the range of values of individual patients larger in the cross-validation cohorts when compared with the discovery cohorts (table 1).

10.1136/jitc-2020-001607.supp3 Supplementary data

**Table 1:** Baseline patient characteristics

	Mel-CTLA4-A	Mel-CTLA4-B	Mel-PD1-A	Mel-PD1-B	NSCLC-PD1
Total, n	10	29	29	36	56
Age, median (range)	59.3 (26 to 79)	58.3 (35 to 86)	64.0 (39 to 84)	61.6 (31 to 83)	63.1 (35 to 81)
Gender, n					
Male	4	13	16	21	36
Female	6	16	13	15	20
Primary tumor, n					
Melanoma	10	29	29	36	0
NSCLC	0	0	0	0	56
Adenocarcinoma	0	0	0	0	37
SCC	0	0	0	0	17
Large cell carcinoma	0	0	0	0	2
Treatment regimen, n					
Anti-PD-1	0	0	29	36	56
Nivolumab	0	0	1	14	50
Pembrolizumab	0	0	28	22	6
Anti-CTLA-4					
Ipilimumab	10	29	0	0	0
Prior therapy lines, n (%)					
0	4 (40%)	–	20 (69%)	30 (83%)	1 (2%)
1	4 (40%)	–	9 (31%)	6 (17%)	46 (82%)
2	2 (20%)	–	0 (0%)	0 (0%)	7 (12%)
>2	0 (0%)	–	0 (0%)	0 (0%)	2 (4%)
Prior immunotherapy, n					
No	10	29	29	36	56
Yes	0	0	0	0	0
Cerebral metastasis, n (%)					
No	6 (60%)	–	17 (59%)	15 (42%)	0 (0%)
Yes	2 (20%)	–	11 (38%)	2 (5%)	0 (0%)
Unknown	2 (20%)	–	1 (3%)	19 (53%)	56 (100%)
LDH (U/L), median (range)	207 (168 to 247)	369 (283 to 881)	229 (124 to 359)	315 (128 to 1523)	269 (133 to 860)

All patients received immune checkpoint inhibitor monotherapy and did not receive any prior line of immunotherapy.

LDH, lactate dehydrogenase; NSCLC, non-small cell lung cancer; PD-1, programmed cell death 1; SCC, squamous cell carcinoma.

### Kinase activity profiles in PBMC of patients responding to checkpoint blockade

**Figure 2:**
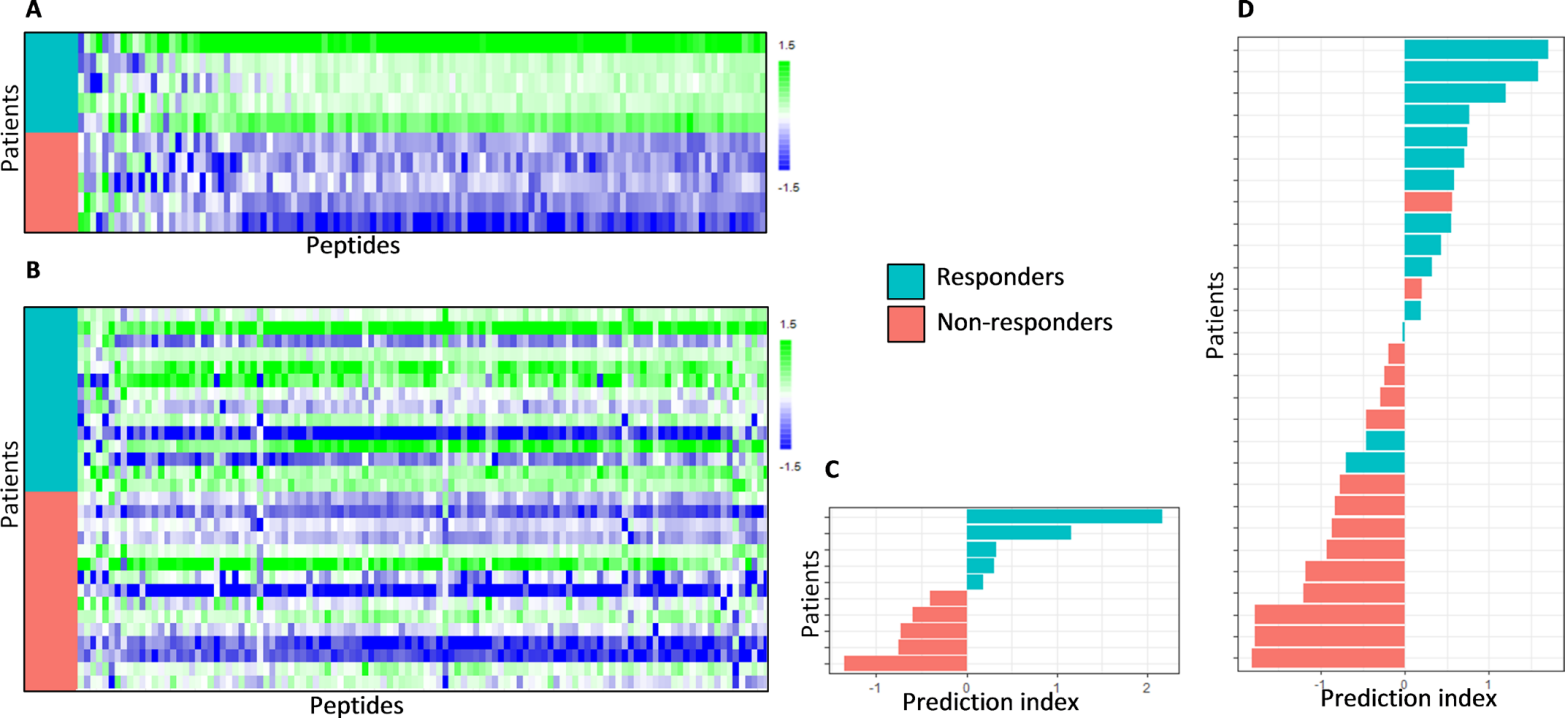
Baseline kinase activity profiles and response classification of patients treated with CTLA-4 blockade. Heat maps showing kinase activity of separate cohorts of melanoma patients who were treated with CTLA-4 immune checkpoing inhibitors. (A) Discovery cohort *Mel-CTLA4-A* and (B) cross-validation cohort *Mel-CTLA4-B*. Binary grouping of patients that either benefited or not from treatment (responders and non-responders, respectively) are shown. The rows represent patients sorted from top to bottom according to treatment response; the columns represent peptides sorted according to Pearson correlation coefficient with treatment response, such that the peptides with a relatively higher phosphorylation signal in the non-responders are shown at the left side of the map and the peptides with a relatively high signal in the responders are shown at the right side of the map. The values are scaled per column to zero mean and unit variance. Furthermore, classification analyzes of these cohorts are shown. (C) Discovery cohort *Mel-CTLA4-A* and (D) cross-validation cohort *Mel-CTLA4-B*. The bar graphs show for each patient the prediction index obtained by cross validation of a partial least square discriminant analysis model (see methods section). If the prediction index is >0, the patient is predicted to be a Responder. The color of the bars indicates the actual clinical response classification.

The correlation of kinase activity profiles with treatment response is visualized for the discovery cohort *Mel-CTLA4-A* using a heat map (figure 2a). A profound difference in kinase activity was observed between the responder and non-responders. Generally, the phosphorylation signal of peptides was higher in responders compared with non-responders. For 83% of the target peptides, a significantly higher signal was found in responders compared with non-responders (two-sided two sample t-test, p value<0.05; FDR<5%). This overall increase in kinase activity was confirmed in the cross-validation cohort *Mel-CTLA4-B* (figure 2b), although less pronounced since only 23% of the target peptides displayed a significantly higher signal in responders compared with non-responders (two-sided two sample t-test, p value<0.05, FDR=18%). In addition, the relative increase in signal was higher in the *Mel-CTLA4-A* cohort (median Log2 fold change=0.84; SD=0.15; ~80% increase) compared with the *Mel-CTLA4-B* cohort (median Log2 fold change=0.40; SD=0.17; ~30% increase). This increase in signal appears to reflect systemic, non-specific signaling in the responders compared with the non-responders.

**Figure 3:**
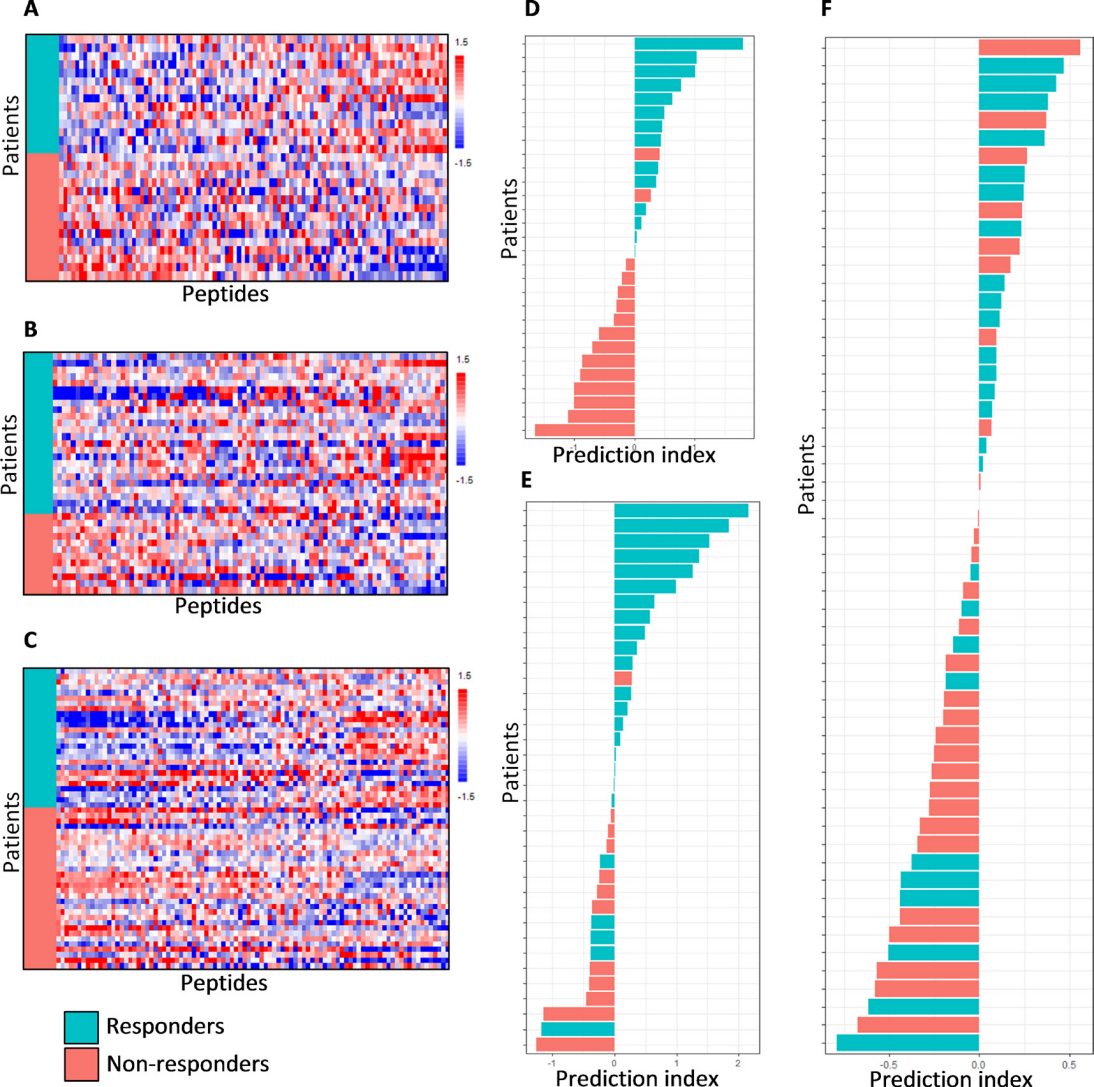
Baseline kinase activity profiles and response classification of patients treated with PD-1 blockade. Heat maps showing the kinase activity of separate cohorts of melanoma patients who were treated with PD-1 immune checkpoint inhibitors. (A) Discovery cohort *Mel-PD1-A*, (B) cross-validation cohort *Mel-PD1-B* and (C) second cross-validation cohort *NSCLC-PD1*. See legend to figure 2 for details. Furthermore, the classification analysis of these cohorts are shown. (D) Discovery cohort *Mel-PD1-A*, (E) cross-validation cohort *Mel-PD1-B* and (F) second cross-validation cohort *NSCLC-PD1*. Again, see legend to figure 2 for details. NSCLC, non-small cell lung cancer. PD-1, programmed cell death 1.

Discovery cohort *Mel-PD1-A* included melanoma patients who were treated with anti-PD-1. The profound higher kinase activities as observed in the responders in the two anti-CTLA-4 cohorts was not observed in the three anti-PD-1 cohorts. Therefore, the data was normalized for overall kinase activity using the VSN method. As a consequence, the data reflects differences in the ratio between peptides on the array rather than the overall differences. Responder patients showed a different kinase activity profile when compared with non-responders (figure 3a). For 17% of the peptides in cohort *Mel-PD1-A*, a significant different signal was found in responders compared with non-responders (two-sided two sample t-test, p<0.05; FDR=29%). These differentially phosphorylated peptides represented both higher and lower signals in responders compared with non-responders. Likewise, differential peptide phosphorylation was observed in the cross-validation cohorts *Mel-PD1-B* and *NSCLC-PD1* (figure 3b–c). In *Mel-PD1-B,* 16 peptides (18%) displayed significantly differential signals for response (two-sided two sample t-test, p<0.05, FDR=25%) whereas *NSCLC-PD1,* 18 peptides (19%) were significantly differently phosphorylated in responders compared with non-responders (two-sided two sample t-test, p<0.05, FDR=24%). The specific differences in kinase activities differentiating responders and non-responders varied between cohorts.

### A high percentage of patients is correctly classified for response by their kinase activity profile

To investigate the potential use of kinase activity profiling as a biomarker for response to ICI therapy, classification analysis was performed using the binary grouping of responders and non-responders. Because of the kinase profile variation between the CTLA-4 and PD-1 ICIs cohorts, a separate PLS-DA classification model was trained for each cohort and predictive scores for each patient were obtained using cross-validation. This resulted in a CCR of 100% (90% CI 74% to 100%) in the discovery cohort *Mel-CTLA4-A* and 83% (64% to 93%) in cross-validation cohort *Mel-CTLA4-B* (figure 2c and d). The CCR was 93% (80% to 99%) in the discovery cohort *Mel-PD1-A,* 78% (63% to 88%) in cross-validation cohort *Mel-PD1-B*, and 68% (56% to 78%) in the second cross-validation cohort *NSCLC-PD1* (figure 3d–f).

### Upstream kinase and canonical pathway analysis identify kinases associated with T-cell function

**Figure 4:**
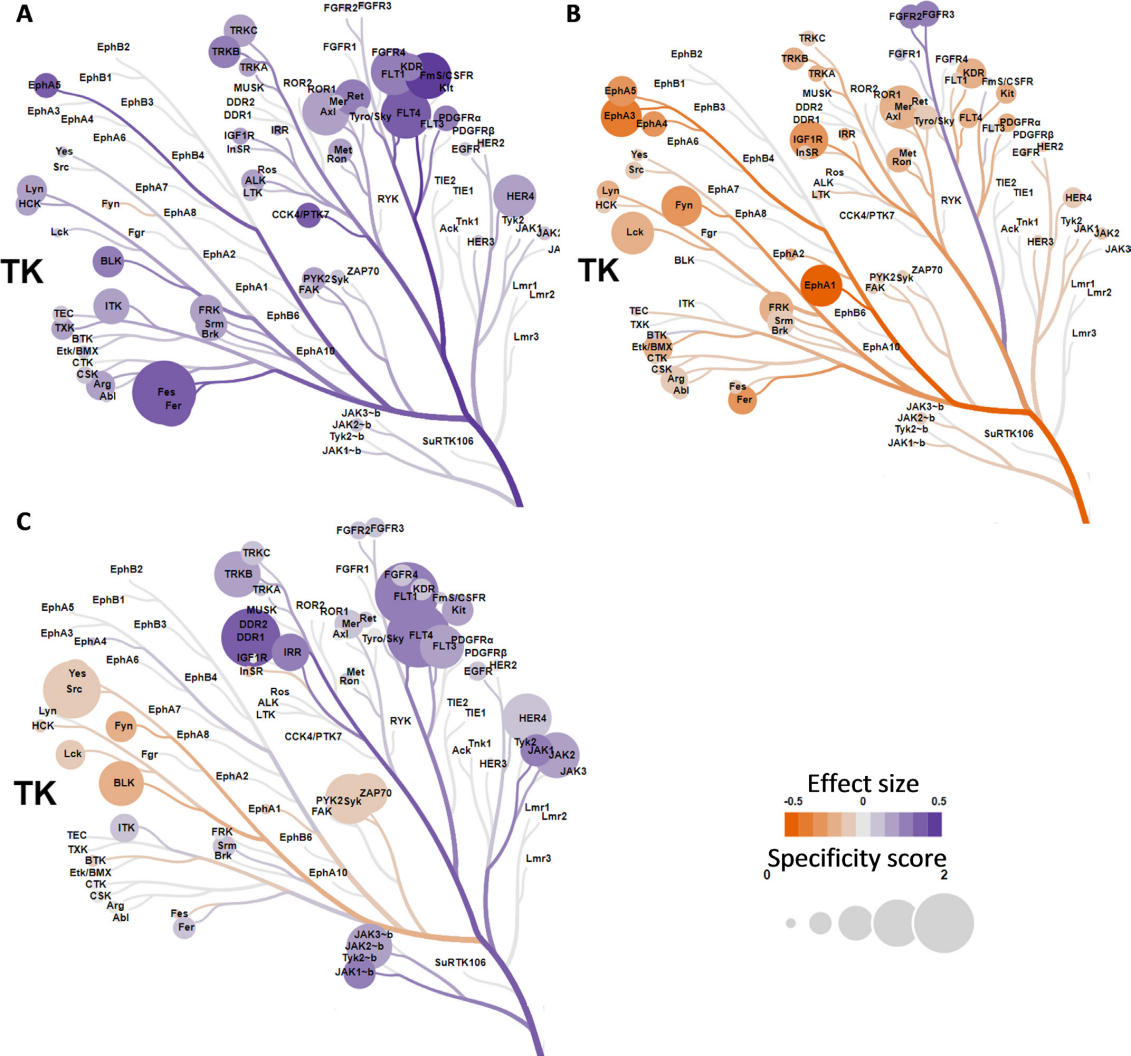
Identification of involved kinase families by phylogenetic tree analysis. Kinase activities that were measured for the anti-PD-1 treated cohorts, *Mel-PD1-A* (A), *Mel-PD1-B* (B) and *NSCLC-PD1* (C) were exposed to the Coral tool to annotate kinases onto a phylogenetic tree (courtesy Cell Signaling Technologies). The coloring indicates the effect size (purple: increased phosphorylation; and orange: decreased phosphorylation in responders compared with non-responders), and the size of the circle indicates the specificity score of the corresponding kinase (a higher score indicates a higher likelihood to contribute to the observed phosphorylation changes). NSCLC, non-small cell lung cancer; PD-1, programmed cell death 1.

**Figure 5:**
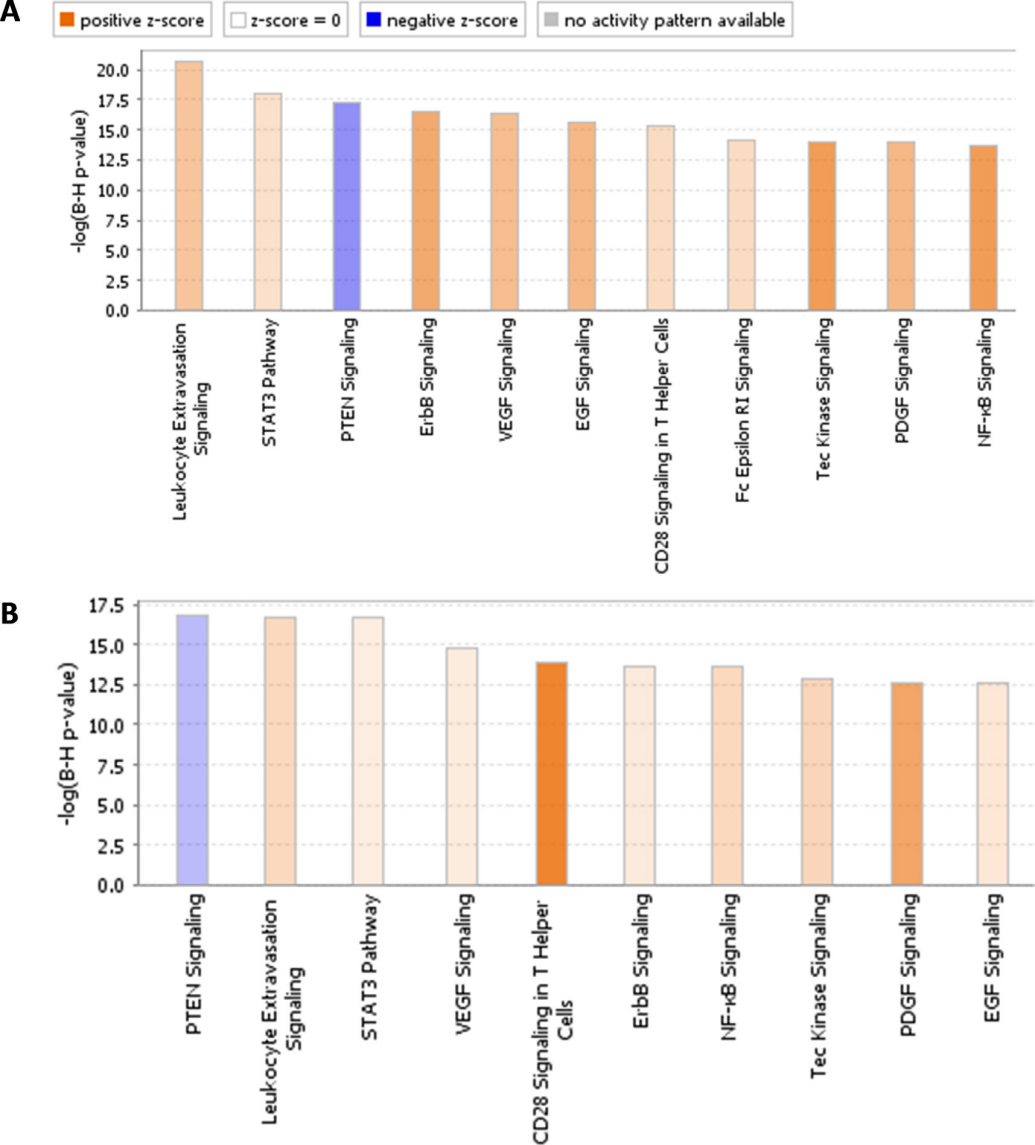
Differential kinase activity involves canonical pathways associated with T-cell function analysis. Top canonical pathways according to kinase activities are schematically shown for cohort (A) *Mel-PD1-A* and (B) *NSCLC-PD1*. Orange (positive z-score) indicates a predicted upregulation of the pathway in patients who benefit from treatment (responders), blue (negative z-score) indicates a predicted downregulation of the pathway in responders. Gray represents canonical pathways without a predicted activity pattern. The significance value indicates the probability that involved kinases are associated with the canonical pathway by random chance alone, cut-off was set at a B-H p value>12. Ranking was based on the trend and z-score. NSCLC, non-small cell lung cancer.

Bioinformatics was applied to interrogate the biological processes underlying response or resistance to ICIs. Here, we have zoomed in on the anti-PD-1 cohorts as the overall increase of kinase activity in anti-CTLA-4 responders hampered proper identification of relevant and recognized peptide targets. Identified kinases were annotated to a phylogenetic tree for protein tyrosine kinases (figure 4). In the discovery cohort *Mel-PD1-A*, predictions revealed that the vascular endothelial growth factor (VEGF) family kinases and feline sarcoma (FES)/FES-related (FER) have relatively higher activity in responders compared with non-responders. Similarly, but less pronounced, the activity of TYRO-3, AXL and MER kinases of the TAM-family and the tropomyosin receptor kinase (TRK)-family were positively correlated with response in this cohort. In the cross-validation cohort *Mel-PD1-B*; however, the activity of several kinases, including the SRC family kinases, were negatively correlated with response. Interestingly, the above observations were corroborated in the second cross-validation cohort *NSCLC-PD1*, where VEGF kinases were found to have higher activity and the SRC family kinases to have lower kinase activity in responders compared with non-responders. Canonical pathway analysis using *Mel-PD1-A* and *NSCLC-PD1* cohorts revealed the importance of peptide targets involved in immune cell migration/leukocyte extravasation and co-stimulation of T helper cells. Finally, increased kinase activity in the STAT3, ERBB, VEGF and EGF signaling pathways were found to be related to response to anti-PD-1, whereas the PTEN activation was associated with resistance to anti-PD-1 (figure 5 and online supplemental figure 3).

10.1136/jitc-2020-001607.supp4 Supplementary data

## Discussion

In this study, we have investigated whether the clinical response to ICIs is reflected by the kinase activity profile in PBMC. We observed differential kinase activity profiles between patients with and without clinical benefit, which were subsequently used to develop a predictive model with a high correct classification rate (68% to 100%) in metastatic cancer patients who were treated with ICI monotherapy.

The predictive power of kinase activity profiling positively compares to currently recognized biomarkers for response to ICIs. For instance, PD-L1 expression in the tumor has a sensitivity and specificity ranging from 58% to 85% and 49% to 60%, respectively, depending on the tumor type, applied cut-off or type of anti-PD-1 antibody.^31^ A similar predictive performance has been reported for TMB.^32^ The kinase activity profile, reported here, demonstrated a lower predictive performance for response to PD-1 inhibitors in NSCLC compared with melanoma. Potentially, this is due to prior systemic treatments since nearly all NSCLC (98%) but only a few melanoma (28%) patients included in this study were pretreated, this may have impacted the outcomes of the kinase profiles.

Responsiveness to CTLA-4 ICIs in melanoma was associated with higher overall kinase activity at baseline in responders when compared with non-responders, suggesting a generally more active immune system in responder patients. Indeed, higher pre-existing T-cell activity has been associated with a better response to CTLA-4 blockade in mouse models.^33 34^ In line with reports on circulating immune-suppressive cells in metastasized patients,^35^ our findings may implicate that immune suppression is less pronounced in responder patients.

The melanoma or NSCLC patients showing a clinical response to PD-1 blockade displayed a more restricted kinase profile. Upstream and canonical pathway analysis of differentially activated peptide targets extend earlier reports on the mechanism of action of PD-1 ICIs. First, high activity of TAM-family kinases, such as TYRO-3, AXL and MERTK, in responders fits findings by others that MERTK becomes activated in CD4^+^ and CD8^+^ T cells downstream of TCR signaling.^36^ Second, identification of pathways, such as extravasation and CD28 co-stimulation, extends outcomes of studies demonstrating the key importance of T-cell recruitment and T-cell co-stimulation in anti-PD-1 responses.^7 37 38^ Thirdly, higher activity in the VEGF pathway in responding patients with melanoma and NSCLC is in line with the finding that VEGF-A enhances the expression of PD-1 by cytotoxic lymphocytes in the tumor microenvironment in a mouse model.^30^ Also, in cancer patients, VEGF is implicated to (in)directly enhance PD-1 expression by intra-tumoral T cells, which may be reflected by higher PD-1 expression on circulating CD4^+^ cells and its association with better clinical outcome after ICI in melanoma.^20^ Finally, we also observed lower activity of the PTEN pathway in responding patients with melanoma or NSCLC. This may seem counterintuitive as loss of PTEN in tumor cells was associated with anti-PD-1 therapy resistance.^39 40^ However, in T-cells PTEN functions as a negative regulator of TCR-signaling. In the absence of PTEN, TCR-mediated activation of T cells is strongly enhanced and thresholds for T-cell activation become less dependent on CD28 co-stimulation.^41^ Taken together, the above four lines of evidence argue that kinase acivities and pathways that are differentially present in melanoma and NSCLC patients who respond to anti-PD-1 reflect the presence of circulating tumor-specific T cells.^38^

Recently, the serine threonine kinase (STK) activity profiles in PBMCs from a small group of 28 metastatic NSCLC patients treated with nivolumab as well as healthy individuals were reported.^10^ Baseline activity of the CAMK family and AGC family was higher in the group of patients with relatively lower survival after PD-1 blockade when compared with patients with longer survival or healthy individuals. The authors suggested that this probably reflects multiple lines of prior systemic treatment (including tyrosine kinase inhibitors (14%) or bevacizumab (39%)). We did not observe differences in these kinase families because we determined the PTK instead of STK activity profiles in PBMC lysates.

Our study has some limitations. Although cross-validation led to correct classification rates that varied from 68% to 100% in separate patient cohorts, the underlying kinase activity profiles were not fully consistent. This may be due to differences in the study populations. For instance, the baseline serum LDH levels showed a greater heterogeneity between patients of cohort *Mel-PD1-B* (median LDH 315 U/L; range 128 to 1523) when compared with cohort *Mel-PD1-A* (median 229 U/L; range 124 to 359). Serum LDH is considered a clinically significant prognostic factor for metastatic melanoma^26^ and is incorporated in the M1 subcategory of current TNM cancer staging protocols.^42^ More importantly, differences in the kinase activity profiles may also be affected by the fact that for this exploratory study we did not apply standardized protocols for PBMC isolation across the patient cohorts, neither was pretreatment of patients with corticosteroids or systemic treatment other than immunotherapy taken into account. We did notice that the overall kinase activity was affected by erythrocyte lysis during PBMC isolation and by the timespan between PBMC isolation and blood collection when EDTA was used as an anticoagulant. Indeed, EDTA by capturing Ca^2+^ may negatively influence PTK activity.^43^ Moreover, considering the relatively large number of kinase spots that were determined in modest patient cohorts, prospective validation with sufficient power is needed before it may be clinically applicable for treatment selection. Finally, the kinase activities point at major involvement of T-cell pathways governing migration, tissue infiltration and co-stimulation. In this study, we used PBMC lysates for kinase activity profiling of individual patients, yet, kinase activity profiling of specific immune subsets may further improve the response prediction and provide more detailed insights in the biological mechanisms of response and resistance to ICIs.

In conclusion, we have demonstrated the potential of kinase activity profiling of PBMCs for response prediction after ICI in separate cross-validation patient cohorts. In a first attempt to address the remaining challenges, the standardization of PBMC isolation protocols and the interrogation of kinase activity in subsets of immune cells has been incorporated in a currently ongoing prospective study to validate the predictive value of kinase activity profiles in PBMCs for ICI response.
