# Supplementary material for: Blood-based kinase activity profiling: a potential predictor of response to immune checkpoint inhibition in metastatic cancer
Source: J Immunother Cancer. 2020 Dec 22;8(2):e001607. doi: 10.1136/jitc-2020-001607 (PMC7757459; doi:10.1136/jitc-2020-001607)
Supplement: Supplementary data [file jitc-2020-001607supp001.pdf]

Supplementary Table 1.

Cohort	ICI	Study site	Anticoagulant	PBMC isolation within	Erythrolysis
Mel-CTLA4-A	CTLA-4	Center A	Na-Hep	4 h	no
Mel-CTLA4-B	CTLA-4	Center B	EDTA	24 h	yes
Mel-PD1-A	PD-1	Center A	Na-Hep	4 h	no
Mel-PD1-B	PD-1	Center C	EDTA	24 h	no
NSCLC-PD1	PD-1	Center C	EDTA	24 h	no

Supplementary Table 1. Site-specific study protocols. Five patient cohorts were evaluated in this study. The cohorts are based on the malignancy (melanoma or NSCLC patients), the type of ICI therapy administered and the center were the samples were collected. Patient number per cohort and anticoagulant used for blood collection are listed. An erythrocyte lysis step was performed during PBMC isolation for cohort Mel-CTLA4-B. *Abbreviations: ethylenediaminetetraacetic acid collection tube (EDTA), sodium heparin (Na-Hep).*

Supplementary Tables 2-6. Datasets generated by kinase activity profiling

Included as separate .txt files.
