# Supplementary figures and images for: Blood-based kinase activity profiling: a potential predictor of response to immune checkpoint inhibition in metastatic cancer
Source: J Immunother Cancer. 2020 Dec 22;8(2):e001607. doi: 10.1136/jitc-2020-001607 (PMC7757459; doi:10.1136/jitc-2020-001607)

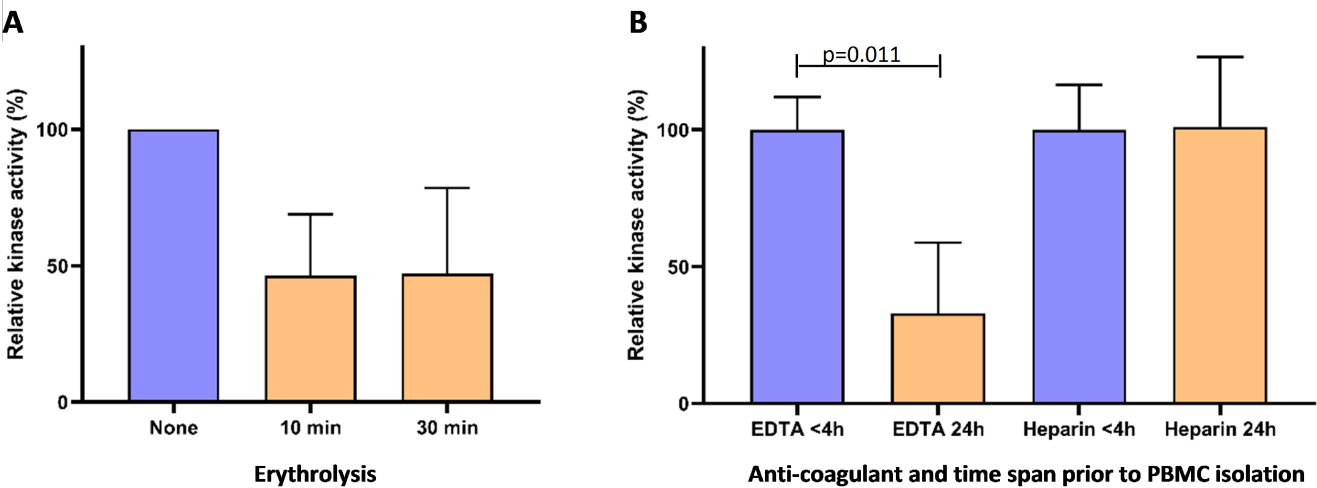

Supplement: Supplementary data [file jitc-2020-001607supp002.pdf]

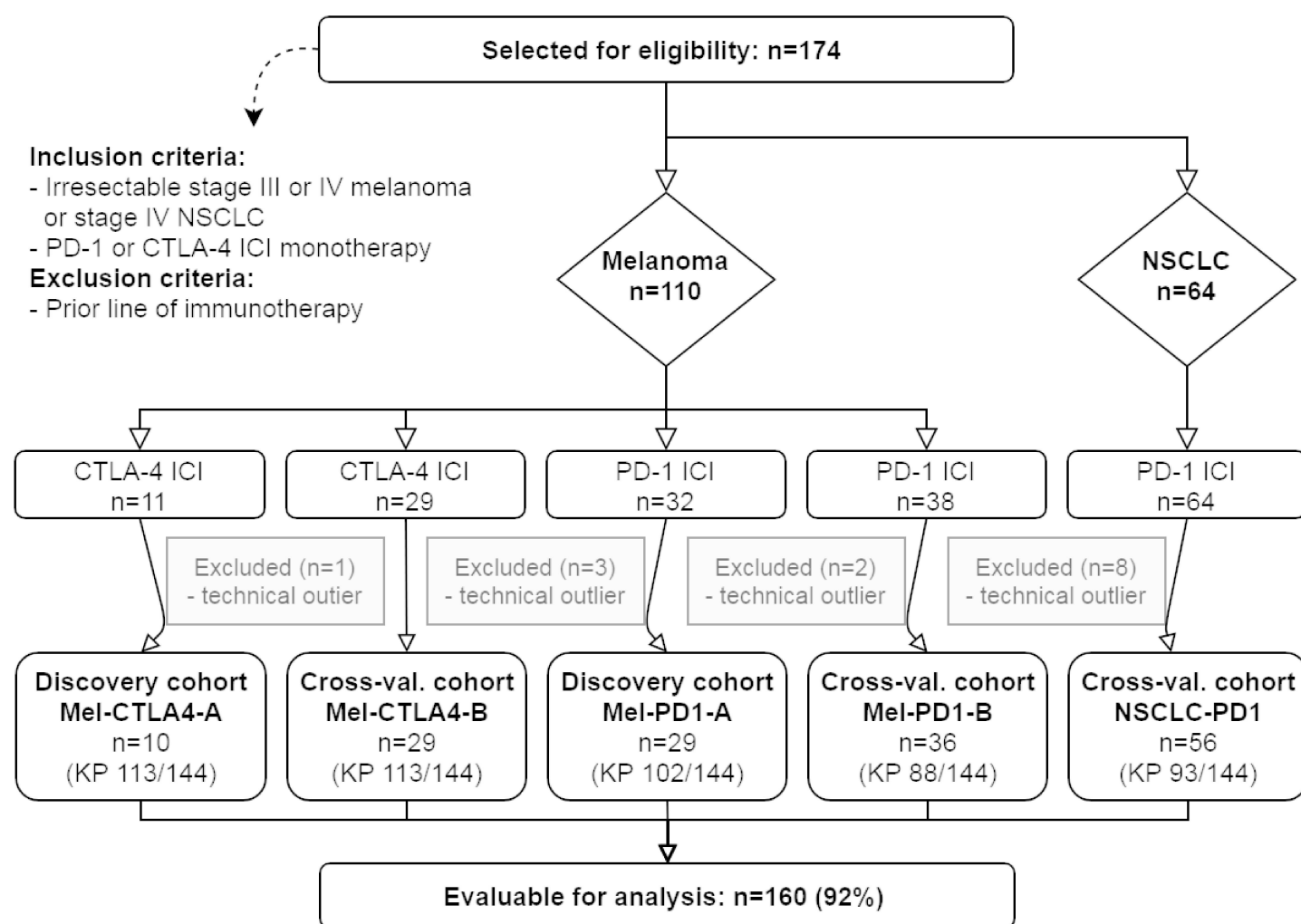

Supplement: Supplementary data [file jitc-2020-001607supp003.pdf]

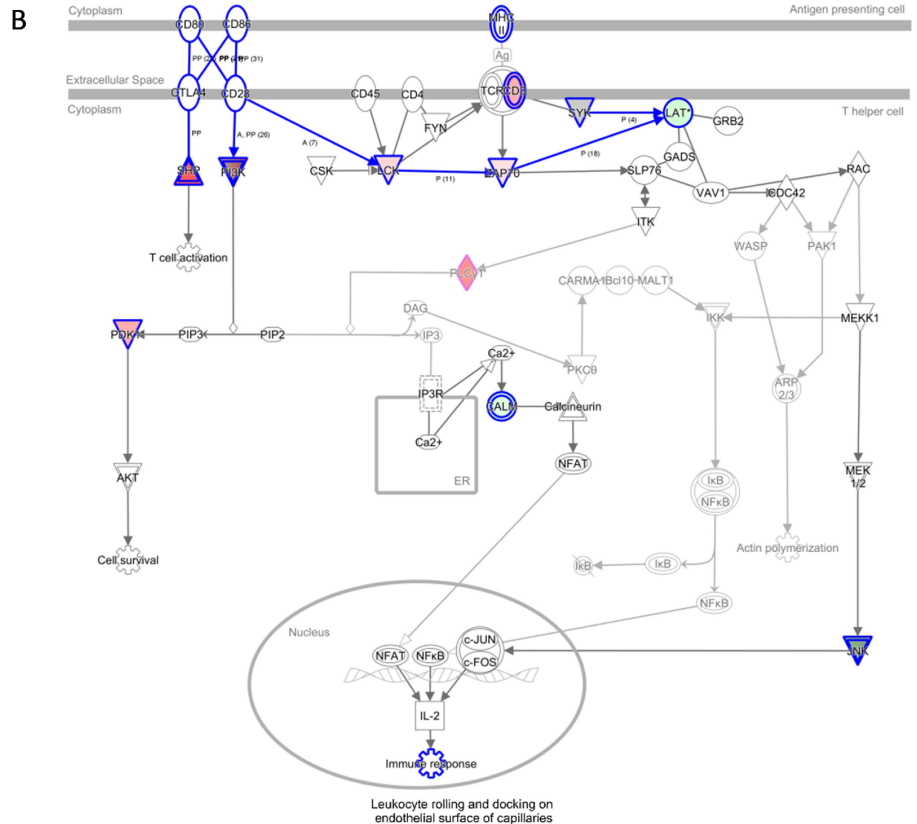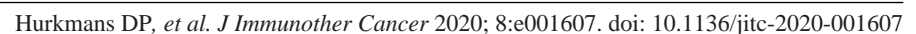

Supplement: Supplementary data [file jitc-2020-001607supp004.pdf]
